# Poisonous Leaves

**Published:** 1882-12

**Authors:** 


### POISONOUS LEAVES.

Some of our most admired flowers, which we should least willingly banish from cultivation are associated with green leaves of a very poisonous character. The narrow, long leaves of the daffodil act as an irritant poison; the delicate compound leaves of laburnum have a narcotic and acrid juice which causes purging, vomiting, and has not unfrequently led to death. The narrow leaves of the meadow saffron, or autumn crocus, gave rise to the utmost irritation of the throat, thirst, dilated pupils, with vomiting and purging. The dangerous character of aconite, or monkshood leaves, is doubtless well known, but each generation of children requires instruction to avoid above all things those large palm-shaped leaves, dark green on the upper surface. Leaves of coarse weeds provide an abundant quota of danger, but frequently their strong scent and bitter or nauseous taste give timely warning against their being consumed. Of all our British orders of plants perhaps the umbelliferous order contributes the rankest and most widespread elements of danger. The tall hemlock is everywhere known to be poisonous, and is one of the most abundant occupants of the hedge. A peculiar "mousy" odor can generally be recognized on squeezing the leaves, which are deep green in color and trebly compound, the small lobes being lanceolate and deeply cut. It is said that the mousy smell can be detected in water containing not more a fifty-thousandth part of the juice. Hemlock is both an irritant to any sore place and a general narcotic poison, producing headache, imperfect vision, loss of power to swallow, and extreme drowsiness, with complete paralysis of voluntary muscles and muscles of respiration. The water dropwort, too, a flourishing ditch plant; the water hemlock, fool's parsley, must be ranked among our most dangerous poisonous plants belonging to the umbelliferous order. The fool's parsley leaves are sometimes mistaken for genuine parsley, but their nauseous odor and darker leaves should prevent this. The nightshade order is another, with dangerous and often extremely poisonous leaves. Indeed, no nightshade can be regarded as safe; while the deadly nightshade, with its oval, uncut leaves, soft, smooth and stocked, is in the highest degree to be avoided. Henbane and thorn-apple, again, with their large and much-indented leaves, are conspicuous members of the "dangerous classes." Holly leayes contain a juice which is both narcotic and acrid, causing vomiting, pain and purging. Even elder leaves and privet leaves may produce active and injurious irritation when eaten. With regard to treatment in cases of poisoning by leaves, if no doctor is at hand, produce vomiting till all offending matter is expelled, and, when considerable sleepiness or drowsiness has come on, give strong tea or coffee, and again bring on vomiting; then stimulate and rouse the brain in every possible mode.—*Land and Water.*

